# Leptin Modulates Exosome Biogenesis in Breast Cancer Cells: An Additional Mechanism in Cell-to-Cell Communication

**DOI:** 10.3390/jcm8071027

**Published:** 2019-07-12

**Authors:** Cinzia Giordano, Luca Gelsomino, Ines Barone, Salvatore Panza, Giuseppina Augimeri, Daniela Bonofiglio, Daniela Rovito, Giuseppina Daniela Naimo, Antonella Leggio, Stefania Catalano, Sebastiano Andò

**Affiliations:** 1 Department of Pharmacy, Health and Nutritional Sciences, University of Calabria, Via Pietro Bucci, 87036 Rende, Italy; 2 Centro Sanitario, University of Calabria, Via Pietro Bucci, 87036 Rende, Italy

**Keywords:** exosomes, breast cancer, leptin, Tsg101, Hsp90

## Abstract

Exosomes—small membrane vesicles secreted by both normal and malignant cells upon fusion of endosomal multivesicular bodies (MVBs) with the plasma membrane—play an important role in cell-to-cell communication. During the last decade, several reports have highlighted the involvement of these nanovesicles in many aspects of breast cancer development and progression, but the extracellular signals governing their generation in breast cancer cells have not been completely unraveled. Here, we investigated the role of the obesity hormone leptin, a well-known adipokine implicated in mammary tumorigenesis, on the mechanisms regulating exosome biogenesis and release in both estrogen receptor α (ERα)—positive MCF-7 and triple-negative MDA-MB-231 breast cancer cells. We found that leptin treatment enhanced the number of MVBs in the cytoplasm of breast cancer cells and increased the amount of exosomes released in cell conditioned media. At molecular level, leptin increased the protein expression of Tsg101—a key component of the endosomal sorting complex required for transport I (ESCRT-I)—by a post-transcriptional mechanism involving its direct interaction with the chaperone protein Hsp90. Targeting leptin signaling, by a selective leptin receptor antagonist the peptide LDFI (Leu-Asp-Phe-Ile), abrogated leptin effects on Tsg101 expression and on exosome secretion in breast cancer cells. In conclusion, our findings, identifying for the first time leptin/leptin receptor/Hsp90 axis as an important regulator of exosome generation in mammary carcinoma cells, suggest that targeting this signaling pathway might represent a novel therapeutic strategy to impair exosome secretion and interrupt the dangerous cell-to-cell communication in breast cancer.

## 1. Introduction

Cell-to-cell communication represents a crucial biological process governing the key events responsible of breast cancer development and progression. Intercellular communication has been considered for long time exclusively mediated by the release of soluble factors that signal through cell surface receptors, by extracellular matrix components or by direct cell-cell contact. However, in the last decades, an additional mechanism represented by the release of membrane-derived vesicles, able to transfer bioactive molecules and signals to both neighboring and distant cells, has been described [1]. Among extracellular vesicles, many evidences suggest that exosomes—nanovesicles of 30 to 150 nm in diameter secreted by endocytic pathway—play an important role in normal mammary gland development and lactation as well as in breast carcinogenesis [2]. The mechanisms of exosome biogenesis/release are deregulated in cancer, and increased numbers of exosomes in cancer cell lines as well as in blood of patients with cancer have been reported [3,4]. In breast cancer, it has been previously indicated that cellular stress condition such as hypoxia [5], and elevated intercellular Ca^2+^ levels might promote the release of exosomes [6]. Exosomes derived by breast cancer cells lead to induction of reactive oxygen species, autophagy and DNA damage repair response in normal human primary mammary epithelial cells, resulting in the creation of a tumor permissive niche [7]. It has also been demonstrated that metastasis establishment and resistance to antitumor therapies can be mediated by horizontal transfer of exosome cargo in recipient sensitive cells. For instance, exosomes from tamoxifen-resistant MCF-7 cells, by transfer miR-221/222, promote tamoxifen-resistant phenotype in MCF-7-sensitive cells [8]. In addition, tumor exosomes might help occurrence of chemotherapy resistance by facilitating drug export outside the cells or delivering P-glycoprotein from docetaxel-resistant cells [9], or might contribute to HER2 target therapy failure by directly binding Trastuzumab and thus interfering with its antitumor activities [10]. Moreover, breast cancer-derived exosomes exert a broad array of effects on tumor microenvironment, by inducing mesenchymal stem cell differentiation into cancer-associated fibroblasts (CAFs) [11], by increasing fibroblast proliferation and lifespan [12], destroying vascular endothelial barriers [13], and impairing the immune response [14,15].

Recent data have been shown that obesity is associated with an increased number of circulating exosomes highlighting a crucial role of these vesicles in the pathophysiology of obesity and its complications [16,17]. Obesity dysfunction is characterized by a reprogrammed adipose tissue associated with an abundant secretion of cytokines, growth factors, inflammatory mediators, along with an altered adipokine profiles [18]. Among the adipokines leptin, whose synthesis and plasma levels increase in proportion to fat mass, has emerged as a key molecule linking obesity to breast cancer [19]. Indeed, we and other authors have extensively demonstrated that leptin, mainly produced by adipocytes but also by epithelial tumor cells itself and by other cells within the tumor stroma (i.e., CAFs) [20], is able to affect several hallmarks of breast cancer [21], impacting directly the phenotype of cancer epithelial cells and indirectly the behavior of the tumor microenvironment [22,23,24]. Here, we described the ability of leptin, the most important adipokine closely related to obesity-associated breast cancer, to influence breast cancer development by modulating exosome biogenesis and release. Particularly, we identified a novel role for leptin in inducing the generation of exosomes from both estrogen receptor-(ER) and progesterone receptor-(PR) positive MCF-7 and in ER-, PR-, and HER2-negative MDA-MB-231 breast cancer cell lines. This occurs at a post-transcriptional level through a leptin-mediated upregulation of Tsg101, a key component of the endosomal sorting complex required for transport I (ESCRT-I), involving the chaperone protein Hsp90 activity. A better knowledge of the signals affecting exosome production in breast cancer cells will increase not only our understanding of breast tumorigenesis and progression, but it might also offer additional possibilities for treating patients with mammary carcinoma.

## 2. Experimental Section

### 2.1. Antibodies and Reagents

Anti-Hsp90 (sc-7947), anti-Tsg101 (sc-7964), anti-GAPDH (sc-47724), anti-Calnexin (sc-11397), anti PARP (sc-7150), and anti-β-Actin (sc-69879) were acquired from Santa Cruz Biotechnology (Dallas, TX, USA); anti-ALIX (EPR14314) was acquired from Abcam (Cambridge, UK); anti-HER-2 (#4290S); antiphosphorylated (p) JAK2 (Tyr1007/1008) (#3776S), -pSTAT3 (Tyr705) (#9138S), and -pMAPK (Thr202/Tyr204) (#9101S); and Rab 4, 7 (#9385), and 27 (#D7Z9Q) were from Cell Signaling Technology (Danver, MA, USA). Leptin and Exosome-Depleted FBS were obtained from Life Technologies (Monza MB, Italy). Acetylthiocholine, 0.1 mM 5,5′-dithio-bis(2-nitrobenzoic acid) (DTNB), and 17-allylamino-17-demethoxygeldanamicin (17-AAG) were purchased from Sigma Aldrich (Milan, Italy).

### 2.2. Cell Cultures

Human MCF-7 and MDA-MB-231 breast cancer epithelial cells (American-Type-Culture-Collection) were stored and authenticated according to supplier’s instructions. MCF-7 cells were cultured in DMEM medium containing 10% FBS, 1% L-glutamine, 1% Eagle’s nonessential amino acids, and 1 mg/mL penicillin–streptomycin at 37 °C with 5% CO_2_ air. MDA-MB-231 cells were grown in DMEM:F12 containing 10% FBS. Primary cell line from mouse mammary tumors, mMTC2 was obtained and characterized as described [25]. mMTC2 was cultured in DMEM supplemented with 10% FBS and 1 mg/mL penicillin–streptomycin at 37 °C with 5% CO_2_ air. Every 6 months, cells were authenticated by single tandem repeat analysis at our Sequencing Core; morphology, doubling times, estrogen sensitivity, and mycoplasma negativity were tested (MycoAlert, Lonza, Basel, Switzerland).

### 2.3. Isolation of Tumor-Derived Exosomes

Cells were seeded at a density of 35 × 10^5^ (MCF-7), 20 × 10^5^ (MDA-MB-231) and 20 × 10^5^ (mMTC2) cells/75 cm^2^ flask in 10 mL of growth medium and then incubated for 48 h in medium containing 10% Exo-depleted FBS. At least 5 flasks/conditions were used. The conditioned medium was harvested and exosomes were isolated by differential ultracentrifugation method [26]. Briefly, media were sequentially centrifuged at 300× *g* and then at 2000× *g* for 10 min to remove large dead cells and cell debris, respectively. The resulting supernatant was further centrifuged at 10,000× *g* for 30 min to remove microvesicles, and the exosomes were pelleted from the final supernatant by ultracentrifugation at 100,000× *g* for 70 min (Sorvall WX Ultra Series Centrifuge, T-865, Thermo Fisher Scientific, Milan, Italy). Finally, the obtained pellet was washed in a large volume of PBS (5 mL) to eliminate any protein contamination and then ultracentrifugated at 100,000× *g* for further 70 min. All steps were carried out at 4 °C. The exosome pellet was resuspended in PBS and stored at −80 °C until use.

### 2.4. Transmission Electron Microscopy (TEM)

Whole exosome extracts were fixed in 2% glutaraldehyde and then absorbed onto formovar-coated grids for 20 min in a dry environment. Cells, after treatment as indicated, were fixed in 3% glutaraldehyde solution in 0.1M PBS (pH 7.4) for 2 h. Then the samples were post-fixed in osmium tetroxide (3%), dehydrated in graded acetone, and embedded in araldite. Ultrathin sections were collected on copper and contrasted using both lead citrate and uranyl acetate. The grids were examined in a Jeol JEM 1400 Plus electron microscope at 80 kV.

### 2.5. Nanoparticle Tracking Analysis (NTA)

The size distribution and the concentration of particles were analyzed using NanoSight NS300 technology (Malvern Panalytical Ltd., Malvern, UK) equipped with a 488 nm laser that utilizes the properties of both light scattering and Brownian motion to obtain measurement of particles in liquid suspension. The assays were performed according to the recommendation of the instrument’s manufacturer. Briefly, two independent replicates of diluted exosome preparation in PBS were passed through the sample chamber. For each sample 10 movies of 60 s were captured and analyzed with Nanosight particle tracking software to calculate nanoparticle concentrations and size distribution. Triplicate measurements were recorded for each sample. Size distribution and concentration profiles were averaged across replicates to derive the size distribution profiles.

### 2.6. Acetylcholinesterase Assay (AchE) to Measure Exosomes

The amount of released exosomes was measured by determining the activity of acetylcholinesterase enzyme following the previously described procedure with minor modifications [27]. Briefly, 50 μL of the exosomal fraction or standard curve samples, prepared using AchE from 0.98 mU/mL to 2000 mU/mL, was transferred into 96-well flat-bottom plates. Samples were then incubated at 37 °C with 1.25 mM acetylthiocholine iodide and 0.1 mM 5,5′-dithio-bis 2-nitrobenzoic acid (DTNB). The change in absorbance at 450 nm evaluated by using AMR-100 (Hangzhou Allsheng Instruments CO., Ltd., Hanghou, China) was monitored every 5 min. The data represent acetylcholinesterase enzymatic activity (mU/mL) after 30 min of incubation.

### 2.7. TUNEL Assay

Apoptosis was determined by enzymatic labeling of DNA strand breaks using terminal deoxynucleotidyl transferase-mediated deoxyuridine triphosphate nick end labeling (TUNEL) using APO-BrdUTM TUNEL Assay Kit (Promega, Madison, WI, USA) as described [28].

### 2.8. Immunoprecipitation and Immunoblot Analysis

Exosomes and cells were lysed in RIPA Buffer (50 mM Tris-HCl, 150 mM NaCl, 1% Nonidet P-40, 0.5% sodium deoxycholate, 2 mM sodium fluoride, 2 mM EDTA, and 0.1% SDS) containing a mixture of protease inhibitors (aprotinin, phenylmethylsulfonyl fluoride, and sodium orthovanadate) for protein extraction. For coimmunoprecipitation experiments, we used 1 mg of total protein extract and 2 µg of Hsp90 or Tsg101 antisera overnight, followed by protein A/G precipitation. Equal amounts of cell and exosome extracts were resolved on 11% SDS-polyacrylamide gel, as described [29]. The bands of interest were quantified by Scion Image laser densitometry scanning program. For a set of experiment, images were acquired using Odissey FC (Licor, Lincoln, NE, USA).

### 2.9. Total RNA Extraction and Reverse Transcription PCR Assay

Total RNA was extracted from MCF-7 and MDA-MB-231 cells using TRIzol reagent and evaluation of gene expression was performed by real-time reverse transcription PCR using a RETROscript kit. The cDNAs obtained were diluted 1:3 in nuclease-free water and 5 μL were analyzed in triplicates by real-time PCR in an iCycler iQ Detection System (Bio-Rad, Hercules, CA, USA) using SYBR Green Universal PCR Master Mix with 0.1 mmoL/L of each primer in a total volume of 30 μL reaction mixture following the manufacturer’s recommendations. Negative control contained water instead of first strand cDNA was used. Primers used for TSG101 were the following: forward 5′-GGAACAATCCCTGTGCCTTA-3′ and reverse 5′-TTTGCATCAACATGCTTTCC-3′. Samples were normalized on 18S (TaqMan rRNA Reagent Kit, Applied Biosystems, Foster City, CA, USA) rRNA content. Relative gene expression levels were calculated as reported [30].

### 2.10. Proximity Ligation Assay

Proximity ligation assay was performed using Duolink Detection Kit (Sigma Aldrich, Milan, Italy) as recommended by the manufacturer. DAPI staining was used for nuclei detection. Fluorescence was detected using a confocal laser-scanning microscope Olympus FV3000 at 40× magnification. Stained Hsp90/Tsg101 complexes were counted by ImageJ software (version 1.51q, NIH, Bethesda, MD, USA).

### 2.11. Statistical Analyses

Each datum point represents the mean ± S.D. of three different experiments. Data were analyzed by Student’s *t*-test using the GraphPad Prism 4 software program. *p* < 0.05 was considered as statistically significant.

## 3. Results

### 3.1. Leptin Induces Generation of Exosomes in Breast Cancer Cells

**Figure 1:**
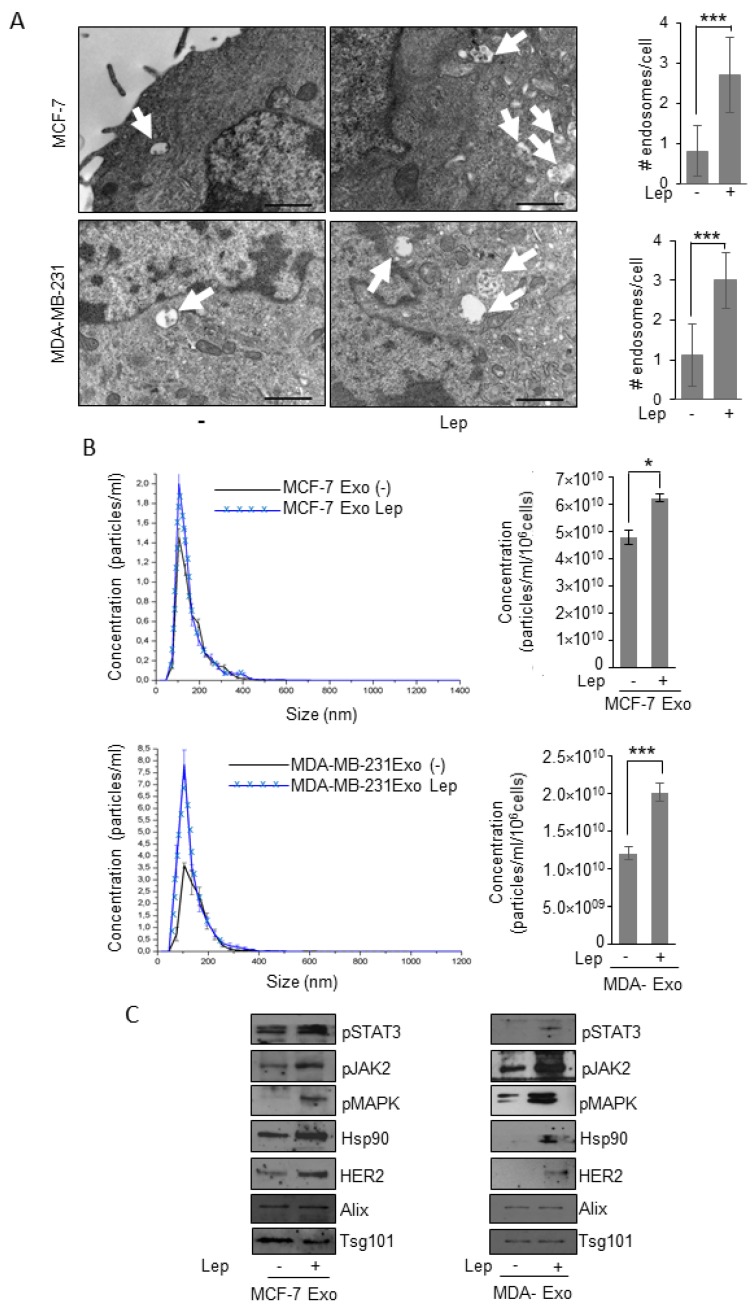
Leptin effects on exosome release in breast cancer cells. (**A**) Representative images of transmission electron microscopy (TEM) showing fields of multivesicular bodies (MVBs) in MCF-7 and MDA-MB-231 breast cancer cells treated with vehicle (-) or leptin (Lep, 500 ng/mL) for 48 h. Scale bar 1 μm. The histograms represent the mean ± S.D. of the MVB numbers in more than 15 fields per condition. (**B**) Representative size distribution profiles of exosomes (Exo), measured by nanoparticle tracking analysis, from conditioned media of MCF-7 and MDA-MB-231 breast cancer cells treated with vehicle (-) or Leptin (Lep, 500 ng/mL) for 48 h. The histograms represent the mean ± S.D. of exosome concentration (particles/mL/106 cells) of three different experiments. (**C**) Immunoblot analysis of pSTAT3, pJAK2, pMAPK, Hsp90, HER2, Alix, and Tsg101 protein expression in exosomal fraction from MCF-7 and MDA-MB-231 treated with vehicle (-) or with Lep for 48 h. * *p* < 0.05, *** *p* < 0.001.

First step in the exosome generation is the accumulation of intraluminal vesicles in late endosome named multivesicular bodies (MVBs). Thus, to investigate the potential involvement of the obesity hormone leptin on exosome generation in breast cancer cells, we evaluated whether leptin may affect these organelles at the ultrastructural level by transmission electron microscopy (TEM) in both ER-positive MCF-7 and triple-negative MDA-MB-231 breast cancer cells. TEM analysis revealed that the number of MVBs in the cytoplasm of leptin-treated breast cancer cells was significantly increased compared to control cells (Figure 1A). To determine if this event might result in an increased number of exosomes released by breast cancer cells, we characterized exosomes isolated in conditioned media of breast cancer cells maintained in the presence of exosome-depleted FBS to avoid the collecting of contaminating vesicles from fetal bovine serum. Isolation of exosomes was carried out by established ultracentrifugation method [26]. The resulting 110,000× *g* pellet representing the exosome fraction was characterized by transmission electron microscopy (TEM) (Figure S1A), which indicated that isolated vesicles were membrane-encapsulated particles with a perfectly rounded shaped morphology, characteristic of exosomes. Moreover, Quantitative Nanoparticle Tracking Analysis (NTA) indicated that the majority of particles, extracted from MCF-7 (MCF-7-Exo) and MDA-MB-231 (MDA-Exo) breast cancer cell conditioned media, were in the expected size range (30–150 nm) to be defined as exosomes (Figure S1B). In addition, immunoblotting assays revealed the expression of classical hallmarks of exosomes, such as Tumor susceptibility gene 101 (Tsg101), and Alix in exosome extracts (Figure S1C). Exosome lysates did not show the expression of the endoplasmic reticulum protein Calnexin (Figure S1C). Hence, the purified particles shared all of the typical features of exosomes. Starting from these experimental conditions, we analyzed the number and the size distribution of vesicles, isolated from the conditioned media of MCF-7 and MDA-MB-231 breast cancer cells treated or not with leptin 500 ng/mL for 48 h, using NTA. As shown in Figure 1B, the concentration of the secreted exosomes in the conditioned medium of leptin-treated cells was significantly higher compared to the number of exosomes seeded from control cells, suggesting that leptin treatment might increase the ability of breast cancer cells to release exosomes. Interestingly, analysis of exosome protein content by immunoblotting revealed that exosomes released from leptin-treated cells contained a larger amount of mediators of leptin signaling including phosphorylated-STAT3 (pSTAT3), phosphorylated-JAK2 (pJAK2), and phosphorylated-MAPK (pMAPK), along with increased expression of the leptin target gene Hsp90 and its client protein HER2 (Figure 1C).

**Figure 2:**
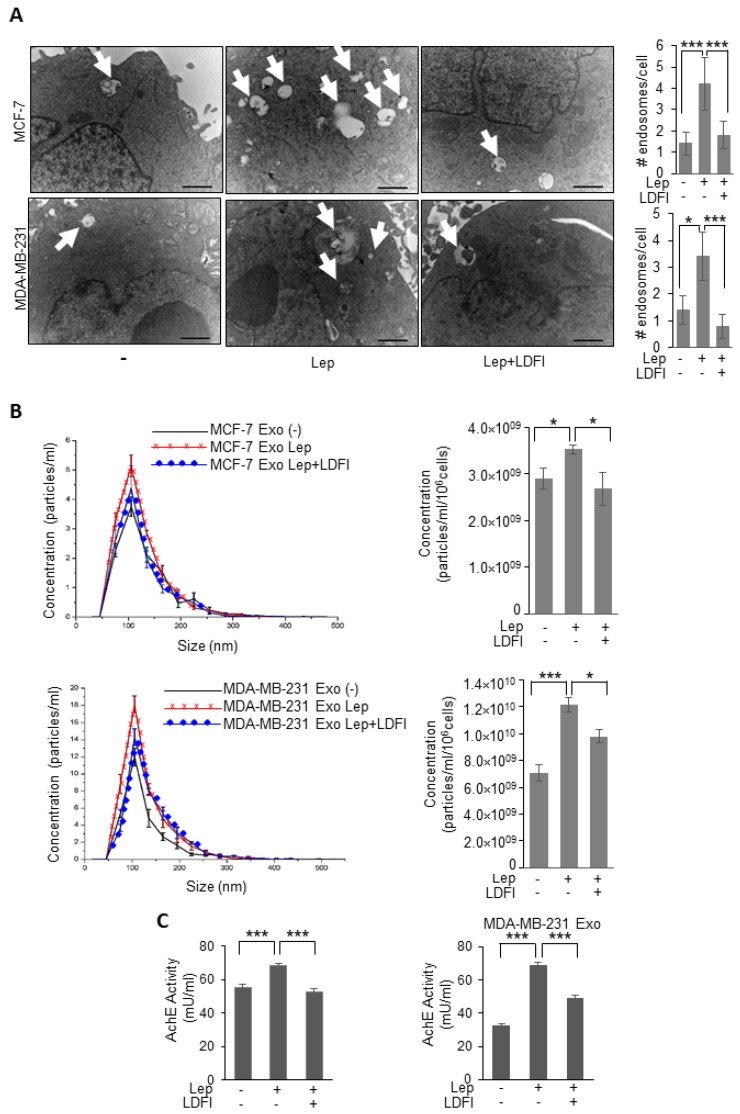
Effects of a selective leptin receptor antagonist (LDFI) on exosome release in breast cancer cells. (**A**) Representative images of transmission electron microscopy (TEM) showing fields of Multivesicular bodies (MVBs) in MCF-7 and MDA-MB-231 breast cancer cells treated with vehicle (-) Leptin (Lep, 500 ng/mL) alone or in combination with the leptin antagonist (LDFI, 1 μM) for 48 h. Scale bar 1 μm. The histograms represent the mean ± S.D. of the MVB numbers in more than 15 fields per condition. (**B**) Representative size distribution profiles of exosomes (Exo), measured by nanoparticle tracking analysis, from conditioned media of MCF-7 and MDA-MB-231 breast cancer cells treated with vehicle (-) or Leptin (Lep, 500 ng/mL) alone or in combination with the leptin antagonist (LDFI, 1 μM) for 48 h. The histograms represent the mean ± S.D. of exosome concentration (particles/mL/10^6^ cells) of two different experiments. (**C**) Concentration of exosomes released from MCF-7 and MDA-MB-231 breast cancer cells treated with vehicle (-), Leptin (Lep, 500 ng/mL) alone or in combination with the leptin antagonist (LDFI, 1 μM) for 48 h measured by acetylcholinesterase activity assay (AchE Activity). * *p* < 0.05, *** *p* < 0.001.

The direct involvement of leptin/leptin receptor mediated signaling in the observed upregulation of exosomes release from breast cancer cells was demonstrated by using a selective inhibitor of leptin activity, the peptide LDFI, a small peptide of the wild type sequence of leptin binding site I, that we recently demonstrated to specifically inhibit both in vitro and in vivo leptin signaling pathway [31]. As shown in Figure 2, in the presence of LDFI peptide, leptin effects on MVB formation and exosome secretion were significantly reduced in both MCF-7 and MDA-MB-231 breast cancer cells, as evidenced by TEM analysis (Figure 2A), NTA (Figure 2B), as well as the measurement of acetylcholine esterase activity (AchE activity), an additional assay used to quantify exosomes (Figure 2C). Moreover, since also apoptotic cells produce microvesicles of diverse size range, we tested if apoptosis might affect our results.

**Figure 3:**
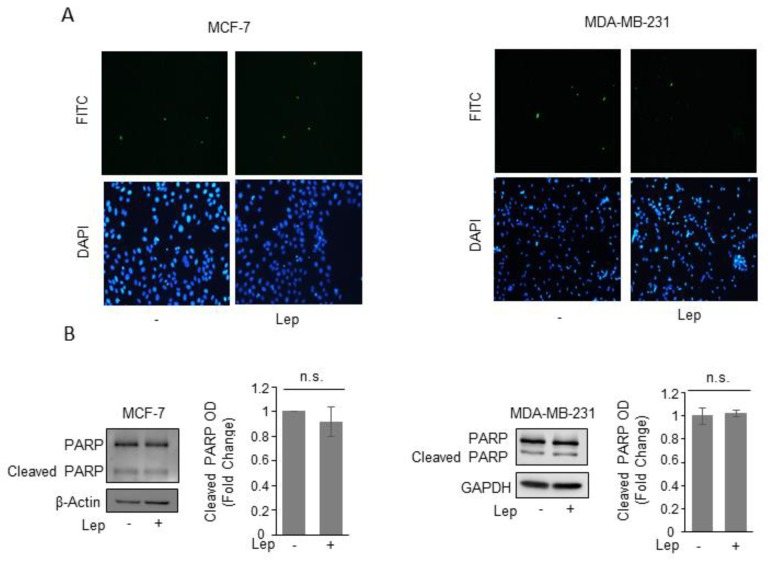
Leptin does not induce apoptotic cell death in breast cancer cells. (**A**) Terminal deoxynucleotidyl transferase-mediated dUTP nick end labeling (TUNEL) staining in MCF-7 and MDA-MB-231 cells treated with vehicle (-) or Leptin (Lep, 500 ng/mL) for 48 h. DAPI was used for nuclear staining. (**B**) Immunoblot analysis of PARP and cleaved-PARP protein levels from total MCF-7 and MDA-MB-231 cellular extracts. β-Actin and GAPDH were used as a control for equal loading and transfer. The histograms represent the mean ± S.D. of three separate experiments in which band intensities were evaluated in terms of optical density arbitrary units (OD) and expressed as fold change versus vehicle-treated samples. n.s. nonsignificant.

We did not find any signs of apoptosis after leptin treatment as demonstrated by the absence of either changes in the internucleosomal fragmentation profile of genomic DNA, evaluated by TUNEL assay (Figure 3A), as well as in the proteolysis of poly (ADP-ribose) polymerase (PARP), a known substrate of effector caspases, by immunoblotting analysis (Figure 3B), in both cell lines.

### 3.2. Leptin Upregulates Tsg101 Expression in Breast Cancer Cells

**Figure 4:**
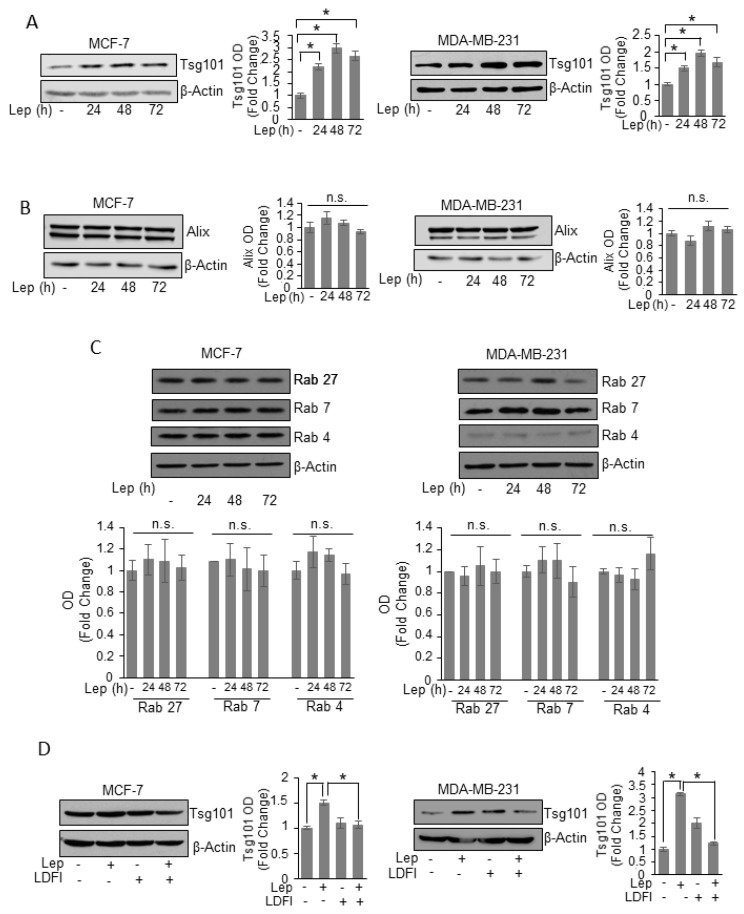
Leptin effects on proteins involved in exosome biogenesis machinery of breast cancer cells. Immunoblots analysis of Tsg101 (**A**), Alix (**B**), Rab 27, Rab 7, and Rab 4 (**C**) in whole cell lysates of MCF-7 and MDA-MB-231 breast cancer cells treated with vehicle (-) or Leptin (Lep, 500 ng/mL) for 24, 48 and 72 h. (**D**) Immunoblot analysis of Tsg101 in whole cell lysate of MCF-7 and MDA-MB-231 breast cancer cells treated with vehicle (-), Leptin and/or LDFI (1 μM) for 48 h. β-Actin was used as a control for equal loading and transfer. The histograms represent the average fold change ± S.D. of three separate experiments in which band intensities were evaluated in terms of optical density arbitrary units (OD), and expressed as fold change versus vehicle-treated samples. * *p* < 0.05, n.s. nonsignificant.

To unravel the possible molecular mechanism by which leptin may modulate exosome formation in breast cancer cells, we performed time course study to evaluate the effects of leptin on the expression of the main proteins involved in exosome biogenesis and secretion processes. We found that leptin treatment significantly increased, at all times investigated, the expression of Tsg101, an important component of the endosomal sorting complex required for transport I (ESCRT-I) involved in the ESCRT-dependent mechanisms of exosome biogenesis [32], in both MCF-7 and MDA-MB-231 breast cancer cells (Figure 4A). In the same experimental conditions, the expression of the ESCRT III complex protein Alix resulted unchanged after leptin exposure (Figure 4B). In the complex mechanisms of the MVBs trafficking to the plasma membrane many proteins of the rab GTPase family are involved [33]. Thus, we also analyzed the expression of components of Rab family proteins, such as Rab 27, Rab 7, and Rab 4.

As shown in Figure 4C, the expression of these Rab family components was not modulated by leptin treatment, indicating that leptin effects on exosome release might be dependent on the induction of Tsg101 expression in breast cancer cells. Finally, the direct involvement of leptin/leptin receptor mediated signaling in the observed upregulation of Tsg101 expression, was demonstrated by using a selective inhibitor of leptin activity, the peptide LDFI. As shown in Figure 4D the presence of LDFI peptide completely reversed leptin effects on Tsg101 expression in both MCF-7 and MDA-MB-231 breast cancer cells.

Interestingly, we found that leptin is able to induce MVB formation, exosome release and Tsg101 expression in primary mouse mammary cell line mMTC2, previously generated in our lab [25] (Supplementary Figure S2), suggesting that leptin-mediated exosome release might represent a general mechanism not related to cell specificity.

### 3.3. Tsg101 Protein Directly Interacts with Chaperon Protein Hsp90 in Breast Cancer Cells

**Figure 5:**
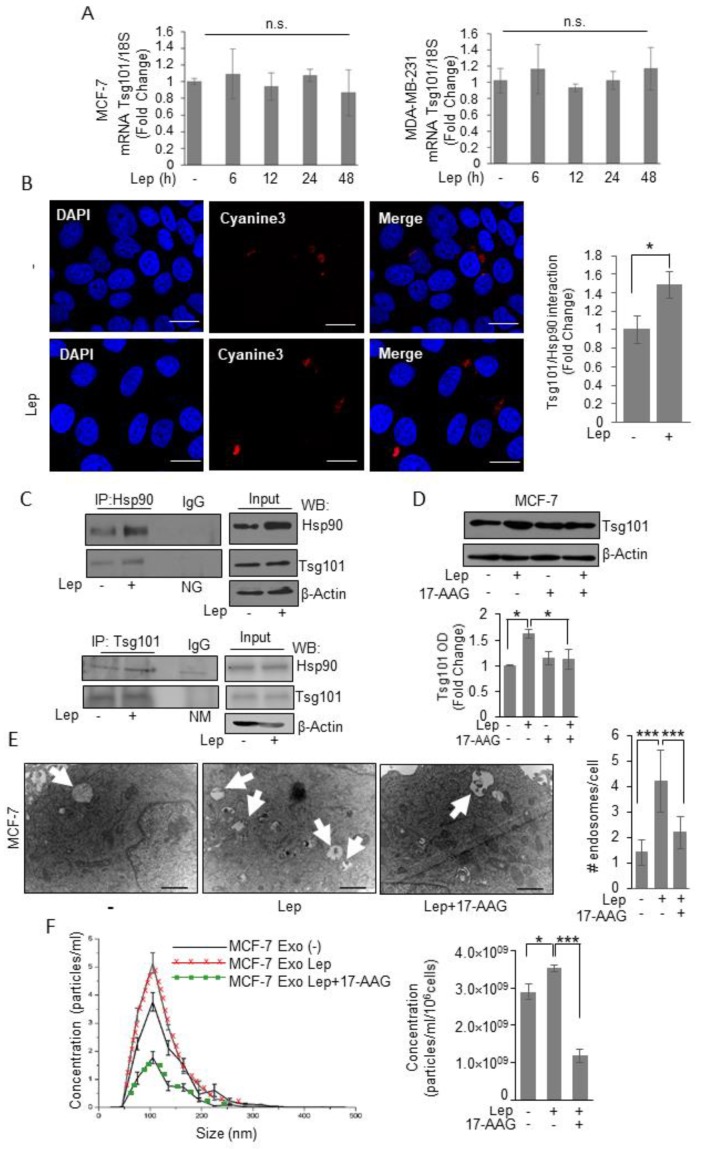
Leptin induces Tsg101–Hsp90 interaction in breast cancer cells. (**A**) Total RNA was extracted from MCF-7 and MDA-MB-231 breast cancer cells treated with vehicle (-) or Leptin (Lep, 500 ng/mL) for 6, 12, 24, and 48 h, then reverse transcribed; cDNAs were subjected to real-time RT-PCR using primers specific for TSG101. Each sample was normalized to its 18S rRNA content. The values represent the means ± S.D. of three different experiments each performed in triplicate. (**B**) Duolink staining (red) was used to detect Tsg101/Hsp90 interaction in MCF-7 breast cancer cells treated with vehicle (-) or Lep for 48 h. DAPI was used for nuclear staining. Red fluorescence was detected by Cyanine3 filter. Images are representative of three different experiments. Scale bar 10 μm. The histograms represent the relative abundance of the Tsg101/Hsp90 complexes (red dots/DAPI) compared to vehicle-treated samples (-) counted by ImageJ software. (**C**) MCF-7 breast cancer cells were treated with vehicle (-) or Lep for 48 h before lysis. Hsp90 and Tsg101 proteins were immunoprecipitated using anti-Hsp90 (IP:Hsp90) and anti-Tsg101 (IP:Tsg101) antibodies respectively and resolved in SDS–polyacrylamide gel electrophoresis. Immunoblotting was performed using anti-Tsg101 and anti-Hsp90 antibodies respectively. Whole-cell lysates were used as input controls. Negative control was performed by incubation of cell lysates with protein A/G agarose and normal goat (NG) or mouse (NM) antisera. (**D**) Immunoblot analysis of Tsg101 in whole cell lysate of MCF-7 breast cancer cells treated with vehicle (-) or leptin alone or in combination with the Hsp90 inhibitor, 17-AAG (20nM) for 48 h. The histograms represent the average fold change ± S.D. of three separate experiments in which band intensities were evaluated in terms of optical density arbitrary units (OD), and expressed as fold change versus vehicle-treated samples. (**E**) Representative images of transmission electron microscopy (TEM) showing Multivescicular bodies (MVBs) in MCF-7 cells treated with vehicle (-) or Leptin (Lep, 500 ng/mL) and/or 17-AAG (20 nM) for 48 h. Scale bar 1 μm. The histograms represent the mean ± S.D. of the MVB numbers in more than 15 fields per condition. (**F**) Representative size distribution profiles of exosomes (Exo), measured by nanoparticle tracking analysis, from conditioned media of MCF-7 breast cancer cells treated with vehicle (-) or Leptin (Lep, 500 ng/mL) alone or in combination with 17-AAG (20 nM) for 48 h. The histograms represent the mean ± S.D. of exosome concentration (particles/mL/10^6^cells) of two different experiments. * *p* < 0.05, *** *p* < 0.001. n.s. nonsignificant.

To explore whether Tsg101 upregulation relies on transcriptional mechanisms, we first evaluated Tsg101 mRNA levels after treatment with leptin for different times. As shown in Figure 5A, we found that leptin treatment did not affect the expression levels of Tsg101 mRNA, at any time investigated. This suggests that a post-translational mechanism could be involved in leptin mediated Tsg101 upregulation in breast cancer cells. Thus, to give a mechanistic explanation of the leptin effect on Tsg101 protein expression, we analyzed specific Tsg101 protein–protein interaction as a possible mechanism able to modulate protein stability within the cells. Particularly, we focused our attention to the possible involvement of the Heat shock Protein 90, a well-known leptin target gene, mainly involved in the control of protein homeostasis as a possible Tsg101 interactor. In situ proximity ligation assay revealed that Tsg101–Hsp90 protein interaction occurred in the cytosol of vehicle treated MCF-7 cells, and the protein complexes appeared significantly increased upon exposure to leptin (Figure 5B). These data were further confirmed by co-immunoprecipitation studies showing a specific interaction between Hsp90 and Tsg101 in basal condition that resulted enriched in the presence of leptin (Figure 5B). Next, to confirm the direct involvement of Hsp90 activity in leptin-enhanced Tsg101 levels, we examined Tsg101 expression upon leptin treatment in the presence of a specific Hsp90 inhibitor, 17-allylamino-17-demethoxygeldanamicin (tanespimycin; 17-AAG). Immunoblotting analysis revealed that in the presence of 17-AAG the leptin-induced increase in Tsg101 expression was completely reversed (Figure 5D). Moreover, we evaluated MVB formation (Figure 5E) and number of exosome released in the conditioned media (Figure 5F) of MCF-7 cells treated with leptin in the absence or presence of the specific Hsp90 activity inhibitor, the 17-AAG. Accordingly, the presence of 17-AAG significantly reversed both leptin-mediated MVB formation as well as the increase in the number of exosome seeded by MCF-7 cells. These data better corroborate the role of leptin-mediated Hsp90 activity in modulating Tsg101 expression, and thus exosome biogenesis in breast cancer cells.

## 4. Discussion

Emerging evidences indicate that exosomes are important contributors to many aspects of breast cancer progression, strongly suggesting the possibility to consider the mechanisms of their generation as new therapeutic targets in cancer cells. Therefore, understanding the extracellular signals governing the inducible biogenesis and release of exosomes from breast cancer cells is imperative for achieving this goal. In this report, we described, for the first time, a novel mechanism by which the adipokine leptin increases exosome generation from both estrogen receptor-positive MCF-7 and triple-negative MDA-MB-231 breast cancer cells. This occurs through an induction of the tumor susceptibility gene 101 (Tsg101) expression, at protein level, mediated by the chaperoning activity of the Heat Shock Protein 90 (Hsp90).

The adipocyte-derived factor leptin has been recognized as an important molecular mediator of obesity in breast cancer [19]. Recent meta-analyses indicated an association between serum leptin levels and breast cancer risk particularly in overweight/obese postmenopausal women [34,35]. Both leptin and its specific receptor are overexpressed in breast cancer tissue, especially in higher-grade tumors, and are associated with distant metastasis [36,37,38,39]. In addition, a growing body of experimental “in vivo” and “in vitro” results clearly demonstrated that leptin exerted several protumorigenic effects, including mitogenesis, transformation, migration, invasion, angiogenesis, and immune cell recruitment [19,21,22]. In this study, we have provided an additional role for this adipokine in breast cancer biology since we have found that leptin is able to regulate exosome biogenesis and release in different models of breast cancer cells. Exosome biogenesis and secretion are very complex mechanisms and a key step in these processes is represented by the accumulation of intraluminal vesicles (ILVs) within large multivesicular bodies (MVBs) that can traffic either to lysosomes for degradation or to the plasma membrane for releasing their cargo into the extracellular space [40]. Our observations at electron microscope level revealed that leptin treatment significantly increased the number of MVBs in the cytoplasm of breast cancer cells and the amount of exosomes released in the conditioned media of cells. Many cellular processes such as apoptosis can influence microvesicles release, but we didn’t find any sings of apoptosis, evaluated by TUNEL assay and PARP cleavage, in cells treated with leptin. Recently, it has also been reported that the endoplasmic reticulum-stress, by inducing the unfolded protein response (UPR), can promote MVB formation and exosome release in different cellular background [41,42]. Thus, we evaluated the intracellular signaling pathways involved in UPR (e.g., Inositol Requiring 1 (IRE1), PKR-like ER Kinase (PERK), and Activating Transcription Factor 6 (ATF6)) after leptin treatment in breast cancer cells without find any activation.

The current knowledge on exosome biogenesis describe different modalities for MVB formation among which the Endosomal Sorting Complex Required for Transport (ESCRT) machinery, responsible for the sorting of ubiquitinated membrane proteins, is the most extensively studied [43,44]. The ESCRT machinery consists of four different complexes, conserved from yeast to mammals—ESCRT-0, ESCRT-I, ESCRT-II, and ESCRT-III—that with sequential actions drive the assembly of cargo in the ILVs within the MVBs [44,45]. Analysis of the functions of different ESCRT components, in major histocompatibility complex class II-expressing HeLa cells, revealed that the inhibition of ESCRT-0/I proteins Hrs, STAM1 and Tsg101 induced a decrease in exosome biogenesis [46]. Moreover, it has been previously demonstrated that depletion of Tsg101 blocks endosomal trafficking [47] and impairs MVB formation in EGF-stimulated cells [48]. Recently, ISGylation-mediated degradation of Tsg101 protein has been reported to be sufficient to impair exosome secretion in HEK293 cells [49], highlighting the fundamental role of Tsg101 in the control of exosome generation. We found that Tsg101 protein expression was significantly enhanced in breast cancer cells treated with leptin, while no differences were found in the expression of Alix, a main component of the ESCRT-III complex. Moreover, we did not observe any significant changes in the expression of proteins belonging to the Rab family of small GTPase, involved in MVB mobilization and fusion to plasma membrane [33], further highlighting Tsg101 as a leptin effector in breast cancer able to control exosome biogenesis. Accordingly, when leptin activity was inhibited by using the full leptin receptor antagonist, the peptide LDFI [31], both Tsg101 expression and the amount of exosomes released by leptin-treated breast cancer cells were reduced. These evidences are in line with the role of leptin in promoting growth and invasion of breast cancer that could be additionally sustained through the induction of Tsg101 expression, since Tsg101 plays an important role in several aspects of breast tumor progression [50]. For instance, downregulation of Tsg101 by small interfering RNA induced apoptosis and inhibited proliferation in MCF-7 breast cancer cells [51], and mammary-gland specific knockout of Tsg101 gene resulted in an impaired growth of epithelial cells as well as in an absence of mammary tumor development [52]. Interestingly, we found that leptin did not affect RNA expression of Tsg101, arising the idea that Tsg101 induction might occurred a post-translational level. One of the most characterized mechanisms involved in the protein homeostasis is the activity of the heat shock proteins (Hsps) that function as chaperones assisting protein folding of several client proteins [53,54]. Although Hsps are traditionally known to be localized and exert their function in the cytoplasmic compartment, many evidences revealed that they can also be localized in the extracellular space and can be secreted by exosomes [55,56,57,58]. A member of the Hsp small family of protein Hsp20 has been demonstrated to regulate Tsg101 expression in cardiomyocytes [59]. Particularly, the authors showed that elevation of Hsp20 promoted exosome secretion by a direct protein–protein interaction with Tsg101, resulting in an activated exosome biogenesis. Accordingly, we found in breast cancer cells that the member of the Hsp family Hsp90, which we have previously demonstrated to be a leptin target gene [60], physically interacts with Tsg101 in basal condition and that the amount of Tsg101 bound to Hsp90 results enriched in cells treated with leptin. Mechanistically, we confirmed that elevation of Tsg101 protein expression as well as induction in the MVB formation and in the number of released exosomes observed in breast cancer cells treated with leptin are dependent on the chaperoning activity of Hsp90, since leptin effects were no longer detectable in the presence of the Hsp90 inhibitor 17-AAG. These results describe leptin/leptin receptor/Hsp90 axis as important regulator of exosome production in breast cancer cells, leading us to include, for the first time, Tsg101 in the big family of Hsp90 client proteins. Of importance, exosomes derived from leptin-treated breast cancer cells display an higher levels of Hsp90, along with other leptin-related molecules such as HER2, pSTAT3, pJAK2 and pMAPK compared to exosomes released from vehicle-treated cells. These results highlight the possibility that an activated leptin signaling in breast cancer cells might not only increase the amount of exosomes released, but it might also change the quality of exosome cargo that could affect neighboring cell phenotypes. This issue deserves more accurate investigations and is currently ongoing in our laboratory.

Overall, our results identify for the first time leptin/leptin receptor/Hsp90 axis as an important regulator of exosome generation in mammary carcinoma cells.

## 5. Conclusions

Many studies have been focused on the identification of specific exosome signatures in attempt to find molecular effectors associated with cancer progression. However, it could be even more imperative deciphering the mechanisms driving cancer exosome production to discover specific targets able to impair exosome secretion and interrupt the dangerous cell-to-cell communication in cancer. In this scenario, our results, increasing the knowledge on the signals involved in exosome biogenesis in breast cancer cells (i.e., leptin signaling), might open new avenues for therapeutic intervention in breast carcinoma, especially in obese women.

## Supplementary Materials

The following are available online at https://www.mdpi.com/2077-0383/8/7/1027/s1, Figure S1: Characterization of exosomes extracted from MCF-7 and MDA-MB-231 breast cancer cells, Figure S2: Leptin effect on exosome release in mMTC2 cells.
